# Genetic variants associated with addictive behavior in Colombian addicted and non-addicted to heroin or cocaine

**Published:** 2013-03-30

**Authors:** Carlos Isaza, Julieta Henao, Leonardo Beltrán, Liliana Porras, Martha Gonzalez, Raquel Cruz, Angel Carracedo

**Affiliations:** a Genetical Medical Laboratory, Pharmacogenetics Research Group, Universidad Tecnológica de Pereira, Facultad de Ciencias de la Salud, Pereira, Colombia, E-mails: caisaza@utp.edu.co; b Institute of Forensic Science, Genomic Medicine Group-CIBERER, Universidad de Santiago de Compostela, Santiago de Compostela, Spain, E-mails: Raquel.cruz@usc.es

**Keywords:** ABCB1 Protein, SLC6A3 protein, heroin addiction, cocaine addiction, c-rack-cocaine

## Abstract

**Objective:**

Determine the prevalence and compare some genetic markers involved in addictive behavior in a group of addicts to derivative of coca (cocaine/crack) or heroin and a control group of non-addicted people matched for gender, age and ethnicity.

**Methods:**

A 120 addicts and 120 non-addicts Colombian male were surveyed and genotyped for 18 polymorphism of the OPRM1, DRD2, DRD4, SLC6A3, SLC6A4, ABCB1, DβH and CYP2B6 genes. For the identification of alleles markers were used mini-sequencing and fragment multiplex PCR techniques; ethnicity of cases and controls was analyzed with 61 AIMs.

**Results:**

The age of onset use of heroin or coca derivatives (cocaine/crack) was 16.5±6 years and 99.2% of them consume several illicit drugs. It showed that controls and addicts belong to the same ethnic group. Significant differences between addicts and controls in relation to schooling, marital status, social security family history of substance abuse (*p *<0.001), Int8-VNTR SLC6A3 gene (*p*= 0.015) and SNP 3435C>T ABCB1 gene (*p*= 0.001) were found.

**Conclusion:**

The present results indicate that the VNTR- 6R polymorphism of the gene SLC6A3 and the genotype 3435CC in the ABCB1 gene, are both associated with addictive behavior to heroin or cocaine.

## Abstract (es)

**Objetivo:**

Determinar la prevalencia y comparar marcadores genéticos involucrados en conducta adictiva en un grupo de adictos a cocaína/crack o heroína y en un grupo control de no adictos apareados por g énero, edad y etnicidad.

**Metodología:**

120 varones adictos y 120 no adictos fueron encuestados y genotipificados para 18 alelos de los genes OPRM1, DβH, DRD2, DRD4, SLC6A3, SLC6A4, ABCB1 y CYP2B6. Para la identificación de los diferentes alelos se utilizaron las técnicas de minisecuenciaci ón y multiplex PCR. La etnicidad de casos y controles fue analizada con 61 marcadores ancestro-informativos.

**Resultados:**

La edad de inicio en el consumo de heroína o derivados de la coca fue de 16.5±6 años y el 99.2% de ellos eran policonsumidores. Los controles y los adictos pertenecen al mismo grupo étnico. Diferencias significativas entre adictos y controles fueron encontradas en relación con escolaridad (*p *<0.001), estado civil (*p* <0.001), seguridad social (*p* <0.001) e historia familiar de drogadicción (*p* <0.001), genotipo int8-VNTR del gen SLC6A3 (*p*= 0.015) y al SNP 3435C>T (rs1045642) del gen ABCB1 (*p*= 0.001).

**Conclusión:**

Los resultados indican que el polimorfismo VNTR-6R del gen SLC6A3 y el genotipo 3435CC en el gen ABCB1, están asociados con conducta adictiva a heroína o cocaína.

## Introduction

Drug addiction is a devastating disease characterized by compulsive search and consumption of drugs, despite their physical, psychological and social consequences. The characterization of the problem and identification of the variables involved in addictive behavior should be a priority in the health plans of any community that suffers the scourge of drug addiction; especially in Colombia, where the problem is critical because this country has had an important role in the production, export, import and consumption of illicit drugs. This situation overflows the field of health and creates serious problems with corruption and violence arising from illicit trade^1^.

The use of drugs with addictive potential can evolve from controlled social use through the abuse, up to the drug dependence, characterized by the search and the compulsive use and dysfunctional. The probability that a person makes the transition from occasional use to addiction depends on factors related to the drug itself (such as availability and toxicological properties), the exposed individual (such as their genetic background and psychiatric morbidity) and the environment (such as family relationships and socio-economic stressors)^2^.Independently of the psychoactive substance used, the addictive disorders share clinical features that suggest the existence of common molecular basis: 1) preferred start in adolescence, chronic course with remissions and exacerbations and strong tendency to relapse; 2) narrowing of behavioral repertoire and continue attitude of seeking and use despite harmful consequences; 3) progressive condition, 4) tolerance; 5) suppression syndrome. The proposed neurochemistry models converge in that all drugs of abuse are addictive because its consumption increased dopaminergic tone in "gratification centers", where after a long period eventually cause adaptations the order biochemical, physiological and structural, involving the mesocorticolimbic system, therefore its proposed that the addiction is caused by an imbalance between the "impulsive" system (limbic system), which governs the search for pleasure, and the "reflective" system (frontal lobe) which executive function is to regulate and control the impulsive system. Chronic exposure to drugs of abuse induces in the patient a neuroplasticity that allows the patient an aberrant form of learning along with a behavior increasingly rigid in terms of search and consumption of the addictive drug, at expense of more adaptive and flexible responses^3^.

Current evidence indicates that polymorphisms of genes encoding the following proteins involved in metabolism, distribution or mechanism of action of cocaine and heroin contribute to both the susceptibility and the response to drugs used in the treatment of addiction caused by these drugs^2^ (a) μ-opioid receptor. Encoded by the OPRM1 gene, mediates the major effects of opioids and some of its polymorphisms have been associated with drug abuse^2^; (b) D2 and D4 dopamine receptors. Dopaminergic stimulation of the brain at critical sites is mediated, among others, these two receptor subtypes. Certain polymorphisms of the DRD2 and DRD4 genes are involved in the abuse of multiple substances, age of onset in the psycho-stimulants abuse and dependence^4^; (c) Dopamine transporter (DAT). The DAT is responsible for active reuptake of DA from the synapse and is the main regulator of dopaminergic neurotransmission in brain regions involved in reward and reinforcement. Pleasurable and addictive effects of cocaine are mediated by the blockage of DAT, and some mutant alleles of the SLC6A3 gene, which encodes this transporter, have been associated with cocaine dependence^5^; (d) Dopamine beta hydroxylase (DβH) The DβH found inside synaptic vesicles of monoaminergic pathways and catalyzes the conversion of dopamine to norepinephrine. DβH polymorphisms appear to influence the effects of cocaine and other stimulants^6^; (e) Serotonin transporter. Serotonin is another neurotransmitter in the CNS actively recaptured from the synaptic vesicle to the presynaptic terminal by its specific transporter, which is encoded by the gene SLC6A4, whose polymorphisms have also been associated with various addictions^7^; (f) P-glycoprotein (P-gp).

Is a key transporter in the disposition of drugs in the CNS, some polymorphisms of ABCB1 gene, which encodes P-gp, produce a poor transporter that modifies the distribution of psychoactive drugs in the CNS and other tissues^8^ ; (g) CYP2B6 enzyme. Among the drug-metabolizing genes, CYP2B6 is one of the most polymorphic and certain allelic variants divide the population into rapid and slow metabolizers of drugs such as methadone, used in the treatment of heroin dependence^9^.

In summary, no conclusive evidence yet pointing to a group of genes as largely responsible for susceptibility to starting of psychoactive drugs and scaling in drug addiction and response to therapies. Identification of these genetic markers will allow early detection of people at risk in order to take appropriate preventive action and offer better treatment options for those who suffer from this lamentable condition. In this study we proposed to determine the prevalence and compare some genetic markers involved in addictive behavior in a group of addicts to derivative of coca (cocaine/crack) or heroin and a control group of non-addicted people matched for gender, age and ethnicity. 

##  Materials and Methods 

### Volunteers:

The case group consisted of 120 men dependent on derivatives of coca (cocaine/crack) and/or heroin, which were internal in 13 rehabilitation centers in Pereira city (Colombia). Inclusion criteria were: 1) age between 16 and 60 years, 2) diagnosis of coca derivatives (cocaine/crack) and/or heroin dependence, according to the DSM-IV, among the cases could be addicted to other drugs of abuse legal (alcohol, nicotine) or illegal, 3) accept the requirements of the study, including the signing of informed consent. Excluded were individuals with chronic neurological disease, cognitive impairment, history of psychiatric illness (dementia or psychosis), epilepsy. The control group was composed of 120 men with no history of drug abuse or dependence of any kind (including alcohol and nicotine), chronic use of psychotropic medication, psychosis, suicide attempts or epilepsy, matched to cases for age, gender and ethnicity. There was not related participants in this study. To avoid problems of population stratification between cases and controls was determined the degree of ethnicity of both groups.

After each participant was informed about the objectives of this study and signed the informed/consent form, oral mucosa sample was taken for genotyping. During personal interview the participants were surveyed and the form designed for the purposes of study, based on the instrument Addiction Severity Index (ASI), was filled. This protocol was approved by the Bioethics Committee of the Faculty of Health Sciences of the Universidad Tecnológica de Pereira (UTP) and rehabilitation centers authorities where patients were treated.

### Single Nucleotide Polymorphism (SNP) Genotyping:

After extraction of genomic DNA obtained from oral mucosa cells, using the Chelex method from FTA cards, we proceeded to the amplification of two fragments of the OPRM1 gene, one of the DβH gene, one of the DRD2 gene, three of the ABCB1 gene and six CYP2B6 gene, in which will be studied including SNP. The PCR fragments was carried out in a total volume of 10 μL, containing 1-10 ng de genomic DNA, 1X Qiagen Multiplex PCR Master Mix (QIAGEN) and 0.2-0.6 μM of each of the primers (Table 1), with following conditions: an initial denaturation step of 95°C for 10 min, followed by 35 cycles of 94°C for 1 min, 60°C for 90 s and 72°C for 50 s, followed by a final extension of 72°C for 7 min. Excess primers and nucleotides were removed by adding 2 μL of ExoSAP-IT (Amersham Pharmacia) according to manufacturer's instructions. Using this pre-amplification product as template, were carried out reactions for the detection of SNPs using the mini-sequencing method (SBE, single base extension). SBE reactions were performed in a total volume of 6 μL, containing 2 μL of pre-amplification product, 2,5 μL of SNaPshot Reaction Mix (Applied Biosystems), 1,5 μL of the SBE primer mixture with concentration of 0,2 μM (Table 1), and amplification for 25 cycles of 96°C for 10 s, 50°C for 5 s and 60°C for 30 s. Excess nucleotide was removed by adding 1 U of SAP (Shrimp Alkaline Phosphatase, Amersham Pharmacia) according to manufacturer's instructions. 1 μL of the SBE reaction product were analyzed by capillary electrophoresis (CE) using the ABI Prims 3100-Avant Genetic Analyzers (Applied Biosystems) with run module SNP36-POP-4. The data were analyzed using the GeneMapper v3.2 software (Applied Biosystems). The presence of the polymorphisms was confirmed by direct sequencing and, ABCB1 polymorphism was confirmed again by Real Time PCR. Variable Number Tandem Repeats (VNTR) and InDel Genotyping. VNTR and InDel typing of the gene DRD4, SLC6A3 and SLC6A4 were conducted in multiplex PCR. PCR fragments was carried out in a total volume of 10 μL, which contain 1-10 ng of genomic DNA, 1X Qiagen Multiplex PCR Master Mix (Qiagen), 1X Q-Solution (Qiagen) and 0,2 mu of each forward and reverse primers of these markers (Table 1).

**Table 1:**
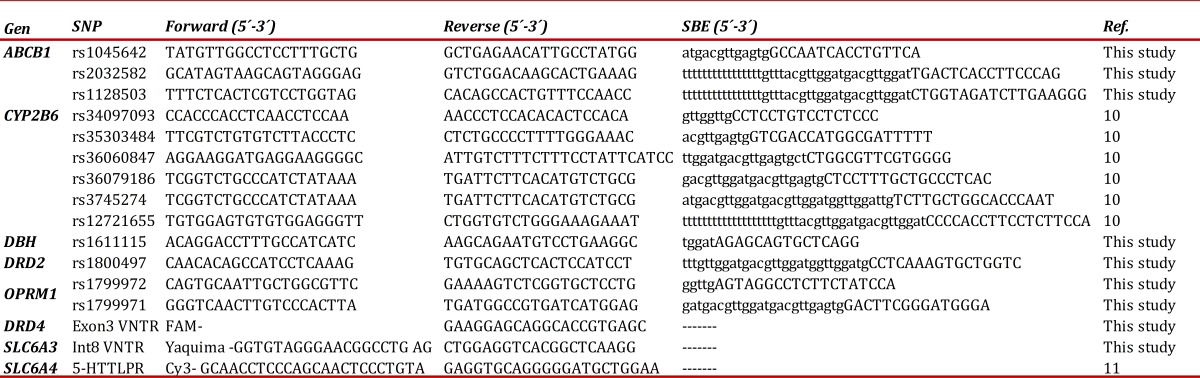
Primers for amplification of fragments and probes for genotyping.

The amplified fragments were carried out with the following parameters: an initial denaturation step for 15 min at 95°C, followed by 40 cycles of 94°C for 1 min, 60°C for 1 min and 72°C for 1 min with final extension at 72°C for 7 min. 1 uL of the amplified product was analyzed by CE using the ABI Prims 3100-Avant Genetic Analyzers (Applied Biosystems) with run module FragmentAnalysis-36-POP-4. The data were analyzed by using the GeneMapper v3.2 software (Applied Biosystems). The presence of the polymorphisms was confirmed by direct sequencing.

### Ancestry-informative markers

(AIMs) Genotyping. 61 markers of ancestry were tested according to the method described by Phillips *et al* ^12^ for discrimination of components European, Asian, African and American ancient. The proportions of single and mixed populations were determined by ancestral populations of the CEPH and finally eingeinstrat and structure analysis of the alleles studied was applied, in order to make the correction of stratification^13^.

### Statistical analysis:

The Chi square test was applied to establish the Hardy-Weinberg equilibrium and comparisons between gene frequencies and the other qualitative variables. Quantitative variables were compared by Student t test.

To determine the combined influence of various factors on the drug addiction was applied multinomial logistic regression model. 95% confidence intervals were used and the level of statistical significance was set at *p* <0.05. For general statistical analysis software was used SPSS for Windows v.19.0; component analysis of ancestry between cases and controls were analyzed using the Structure software 3.1.3, and the R software, with the application engeinstrat.

## Results

### Demographic and clinical variables.

There were no differences between the two groups with respect to age in years (controls: 31±10 vs addicts: 31±10,3, *p*= 0.96), but in relation to the level of education (≤ 11 / ≥ 12 years) (controls: 40/80 vs. 107/13, *p *<0.001), marital status (married, cohabiting / separated-single) (controls: 46/74 vs. 24/96, *p *<0.001), right to security social (yes / no) (controls: 113 / 7 vs 84/36, *p *<0.001) and family history of substance abuse (yes / no) (controls: 43/77 vs. 97/23, *p *<0.001). The age of onset in the consumption of coca derivatives or heroin was 16.5±6 years (range 9-40 years), although it should be noted that almost all addicts had first experienced with other psychoactive; 74.1% was addicted to cocaine derivatives, heroin 4.2% and 21.7% for both drugs, in addition 99.2% (119/120) of them used other illicit drugs, mainly marijuana, amphetamines and tranquilizers. Heroin use was concentrated in younger people (20±1.5 years), crack in older (36±10 years) and cocaine in an intermediate (25±7 years).

### Ancestry-informative genotyping:

According to data from the population structure the ancestry component for cases and controls are Africa (0.1137 vs 0.1297), Europe (0.5268 vs 0.5308), East Asia(0.0100 vs 0.0110) and Native American (0,3495 vs 0,3284); no significant difference (p=0,98) among cases and controls was found.

### Genetic markers of drug addiction:

The SNPs rs35979566, rs35303484, rs28399499, rs36060847, rs12721655, rs34097093 and rs36079186 of CYP2B6 gene, rs1799972 of OPRM1 gene and rs2032582 of ABCB1 gene were monomorphic in the studied samples. The Hardy-Weinberg equilibrium was confirmed with each of the genotypes of addicts and controls, except with the ABCB1 gene rs1128503 in the control group, so this marker was excluded from the analysis. The samples subjected to quality control by sequencing coincided with the previously assigned genotype. According to the allelic and genotypic distributions shown in Table 2, there were no significant differences between addicts and non addicts with respect to the studied polymorphisms of the OPRM1, DβH, DRD2, DRD4, SLC6A4 and CYP2B6 gene. By contrast, between addicts and controls significant associations were found for Int8 VNTR SLC6A3 gene (*p*= 0.015, OR = 4.7 (1.34 -16.2)) and 3435C>T (rs1045642) ABCB1 gene (*p*= 0.0002, OR=2.4 (1.4 - 4.1)). These differences persisted when comparing separate consumers from coca or heroin (data not shown). However, for the Int8 VNTR in SLC6A3, the comparison of the 5R/5R genotype versus all the others displayed was not statistically significant after the Bonferroni correction for multiple testing (*p* =0.16)

**Table 2:**
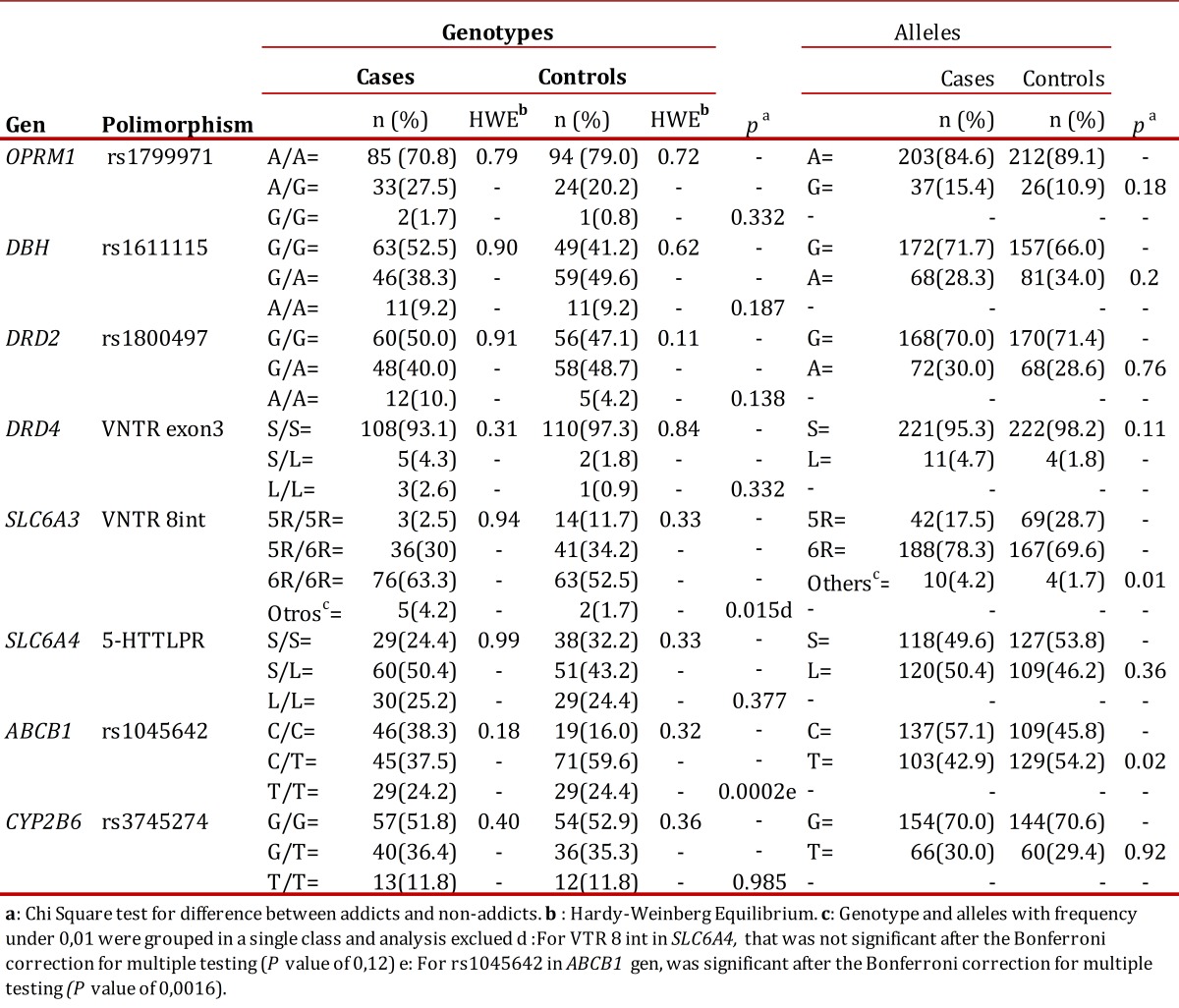
Genotypic and allelic distribution between addicts to heroin or coca derivatives and health controls

The combined influence of various factors on drug addiction was assessed by multivariate analysis, using the condition of addict/control as the dependent variable, and as covariates those that were significant in the bivariate analysis. According to the results shown in Table 3, the low level of education, separated-single marital status, the absence of social security, positive family history drug, carrier status of 6-R allelic variants of SLC6A3 gene and 3435CC genotype of ABCB1 gene, respectively, remain as independent factors associated with addiction heroin and/or derivatives of coca (cocaine or crack) in the patients in our study.

**Table 3:**
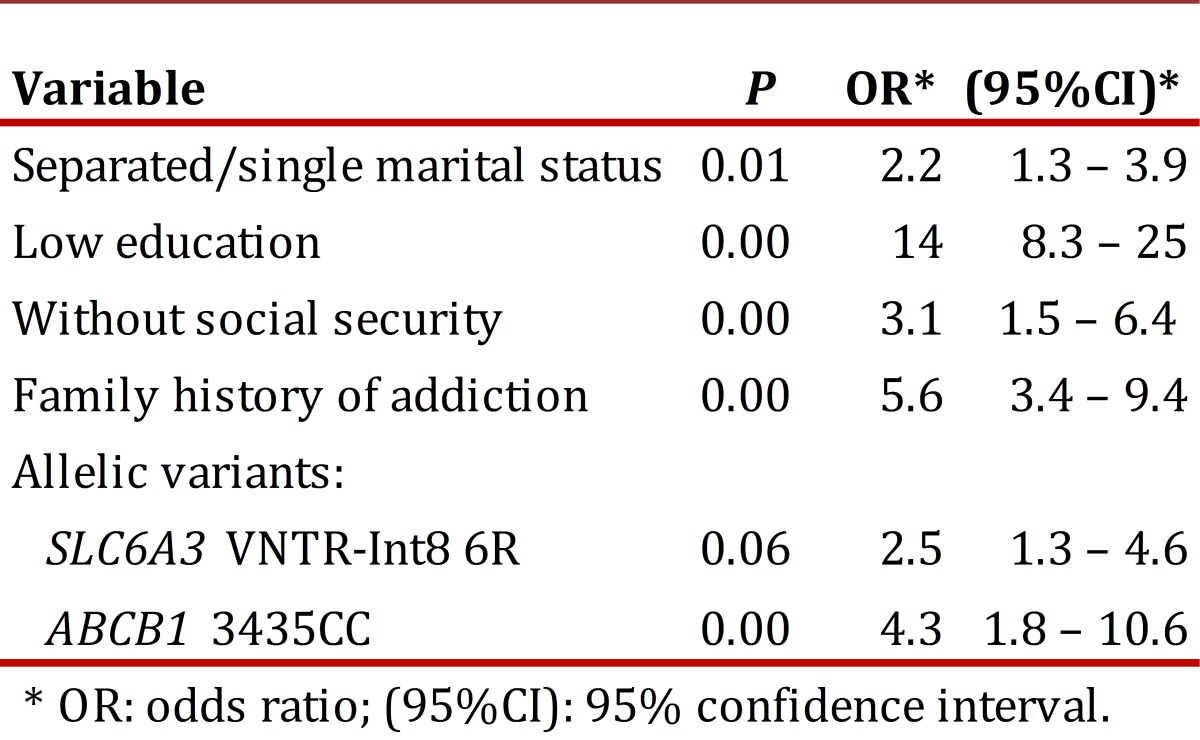
Logistic regression analysis of associated variables with addiction to heroin and/or coca derivatives.

## Discussion

Drug addiction is a multifactorial disease affected by psychological variables, physiological, pharmacological, genetic and environmental. A great deal of current research focuses on the search for factors that influence the vulnerability for the acquisition of addiction, for their persistence and propensity to relapse, which will result in a better scientific basis of prevention and treatment programs for drug addicts. According to the National Survey of Substance Abuse in Colombia (2008), last year 2.7% of surveyed (about 540,000 people) used illicit drugs. Being marijuana and cocaine/crack the most widely used. Respect to heroin, its abuse in the country is more recent and less known, the data suggest that this is already a social problems, especially if one considers that when a person starts their consumption can escalate into abuse and after the drug dependence more rapidly and intensely than for any other illegal drug. It is estimated, for example, that one in four people who try drugs end up addicted and the mortality among heroin users is 10-20 times higher than non-users matched for age and gender, and 12 times higher than the general population^2^.

Many clinical and demographic findings of this study are consistent with several epidemiological reports of heroin and/or coca derivatives such as age of onset (16.5±6 years), high proportion of individuals with a family history of drug abuse in first and second degree (80.8%), without a stable relationship (80%) and without social security (70%). In effect, numerous studies show that most people have become involved in the consumption of psychoactive substances adolescence early, when significantly increases the probability of risky behavior, including experimentation with psychoactive substances. On the other hand, personality disorders, stressful life events and emotional relationships negative, including parent-child relationships are predictors of addictive behavior^14^. In addition, although this study did not want to identify patterns of interaction between genes and environment, should not ignore the abundant evidence on the role of these interactions in the development of addictive behaviors and psychiatric disorders. 119 of the 120 addicts in this study said they consume more than one psychoactive drug, this confirms that the abuse of various drugs is the rule between consumers. Although this study also explored the psychiatric comorbidity, it is good to remember that psychiatric disorders are also very high among drug addicts, the most common mood disorders, posttraumatic stress and antisocial personality. 

As all addicts included in this study were residents of therapeutic communities and participated in rehabilitation programs based solely on abstinence, we should make some comments. Due to a better understanding of physiopathologic mechanisms of addictive disease, it is clear that no single therapeutic approach able to cover its many facets, is necessary multidisciplinary management, with two main therapeutic tools currently available: the pharmacological and psychosocial therapy.

The latter is the therapy of choice for cocaine dependence, but many patients do not respond, the discovery of useful medicines is an emergency, so it has boosted the development of promising drugs that are currently in clinical trials. On the contrary, without ignoring the importance of psychosocial treatment, the drug of choice is the cornerstone of treatment for opioid dependence. So the "maintenance treatment" (suspension of heroin and substitute prescribed an opioid, usually methadone) should be prioritized, not the "detoxification" (stopping the drug, without replacement)^15^. From the point of view neurobiochemistry some neural circuits and neurotransmitter systems are important in the predisposition, development and maintenance of addictive behaviors. Is considered a risk factor for psychoactive substance abuse, the hypodopaminergic status in the reward circuitry of the limbic system, caused by some polymorphisms of genes related to dopamine^6^ ^,^ ^18^ ,some authors speak of the reward deficiency syndrome, RDS, in which the drug addiction predisposition of individuals explained by: high rates of uptake of dopamine (DAT), high rate of degradation of dopamine (DβH) or low levels of dopamine receptors (DR)^16^.

The allele int8-6R of VNTR in SLC6A3 gene is found in greater proportion in the group of drug addicts in this study (Table 3) which confirms this hypothesis can be explained as a phenotype with low dopaminergic tone tends to be offset by the use drugs that stimulate the reward system. Although this result agrees with others^17^,there is also evidence to the opposite result15 or no association between this polymorphism and heroin addiction or alcohol^18^. On the other hand, the 3435CC genotype of gene ABCB1 is associated with expression adequate levels of P-gp, the transporter that acts as a pump of exclusion and plays a key role in the disposition of xenobiotics in the CNS and other organs. It has been reported the association of the 3435C native allele with marijuana dependence^19^ and the effects of a variety of neurological and psychiatric drugs, among which are some opioids^2^.

However, it is unclear whether cocaine or heroin are substrates of P-gp and, to our knowledge, the influence of ABCB1 polymorphisms on the brain disposition to cocaine or heroin has not been investigated so far. Some hypotheses may explain why 3435CC genotype prevalent among drug users in this study; first, psychoactive drugs in the CNS of controls with the mutated allele (phenotype P-gp deficient) produces excessive and dysphoric levels of dopamine, functioning as a protective factor against abuse of psychoactive substances, similar to the action of disulfiram in cocaine addicts. Second, the increased efficiency of the transporter (P-gp competent phenotype) between wild genotype addicts cause euphoric effect of shorter duration, which produces a rapid abstinence syndrome and greater compulsion to continue use. The two hypotheses are not mutually exclusive, but both start from the premise that both cocaine and heroin are substrates of P-gp, which has not been shown yet. Allele rs1800497 (Taq1A G>A) of DRD2 gene results in a lower number of D2 receptors and one type of receptor with lower binding capacity of dopamine^20^.

In this and other studies no relationship between this polymorphism and drug dependence^21^ were found. However most of the studies support an association of the mutated allele A with heroin abuse, alcohol, smoking and various aspects of addictive behavior^21^. The VNTR polymorphism of the DRD4 gene, coinciding with the results of this study, some authors have found no differences of allele S (2 to 6 repeat) and L (7 or more repeat) among drug addicts and controls^21^. However, after a thorough review of scientific literature, McGeavy J ^22^ concluded that there was consistent evidence of the relationship of VNTR allele L with addictive behavior, explained by the fact that VNTR-L allele is associated with lower density receptors and reduced sensitivity to dopamine. Allele rs1611115 (-1021C> T) DβH gene decreases the activity of the enzyme^20^, but evidence of the association of this polymorphism with addictive behavior is weak^5^. Several studies have reported positive association of allele rs1799971 (118 A>G) of OPRM1 gene, encoding the μ opioid receptor, with heroin and alcohol addiction^3^, although in other research, including this, have not replicated these findings^23^. Also in our study significant differences in 5-HTTLPR genotypes S/S, S/L and L/L SLC6A4 gene between addicts and controls were found. For this marker the results are also contradictory because while in a study the SS genotype was associated with risk of abuse of cocaine^24^, other authors have reported a positive association of the variant L with various addictions^7^.

Is necessary to emphasize some limitations of this study. First, the group of addicts studied does not represent all drug addicts, as it only included over 16 years, all of them had sought help and were interned in rehabilitation centers. Second, recognizing that gender is an important factor in substance abuse studies^14^ ^,^ ^25^, in this study, for logistical reasons, only males were included, which excludes the possibility of finding gender-specific associations. Also cannot exclude the possibility of associations between other polymorphisms of the genes studied and addictive disease, as this would require the complete sequencing of genes. It should also be recognized that some genetic markers reported in this study have also been linked with mental illness, which often coexist with drug addiction; this opens the real possibility that the association of genetic polymorphisms is found with some underlying mental illness and not drug addiction. Equally is possible that other genes in linkage disequilibrium to those in this study are those actually associated with drug addiction. Discrepancies in the results of genetic studies related to drug addiction should not be surprising. The discrepancies in the results of genetic studies related to substance abuse are common. This is due largely to the difficulty in identifying risk biomarkers of disease as the drug dependence, result of complex interactions of genetic and environmental factors, which probably involves multiple genes and each of them makes only a modest contribution to total risk^21^. Consequently, the sample size limits the statistical power to detect small contributions from some markers studied. Finally we must recognize that the status of controls was established by self-report and therefore some people in the control group could have hidden or underestimated exposure to substances of abuse. Our findings provide preliminary evidence of the association between the ABCB1 polymorphism and heroin or cocaine dependence and the association between SLC6A3 and heroin or cocaine dependence was confirmed. These findings encourage future efforts in the search for functional polymorphisms in and near the ABCB1 gene using a systemic approach in a larger sample.
